# New Alternatives for Autoimmune Disease Treatments: Physicochemical and Clinical Comparability of Biosimilar Etanercept

**DOI:** 10.1155/2016/9697080

**Published:** 2016-06-12

**Authors:** Mariana P. Miranda-Hernández, Carlos A. López-Morales, Francisco C. Perdomo-Abúndez, Rodolfo D. Salazar-Flores, Nancy D. Ramírez-Ibanez, Nestor O. Pérez, Aarón Molina-Pérez, Jorge Revilla-Beltri, Luis F. Flores-Ortiz, Emilio Medina-Rivero

**Affiliations:** ^1^Unidad de Investigación y Desarrollo, Probiomed S.A. de C.V., Cruce de Carreteras Acatzingo-Zumpahuacán, 52400 Tenancingo, MEX, Mexico; ^2^Dirección Médica, Probiomed S.A. de C.V., Avenida Ejército Nacional No. 499, Colonia Granada, Delegación Miguel Hidalgo, 11520 Mexico, DF, Mexico

## Abstract

Etanercept is a recombinant fusion protein approved for the treatment of TNF-*α* mediated diseases such as rheumatoid arthritis (RA), psoriasis, psoriatic arthritis, and ankylosing spondylitis. Herein, we present an evaluation of the physicochemical and biological properties of a biosimilar etanercept and its reference product followed by a clinical study in patients diagnosed with RA intended to demonstrate comparability of their immunomodulatory activity. Identity analyses showed a total correspondence of the primary and higher-order structure between the two products. In regard to intrinsic heterogeneity, both products showed to be highly heterogenous; however the biosimilar etanercept exhibited similar charge and glycan heterogeneity intervals compared to the reference product. Apoptosis inhibition assay also showed that, despite the high degree of heterogeneity exhibited by both products, no significant differences exist in their* in vitro* activity. Finally, the clinical assessment conducted in RA-diagnosed patients did not show significant differences in the evaluated pharmacodynamic markers of both products. Collectively, the results from the comparability exercise provide convincing evidence that the evaluated biosimilar etanercept can be considered an effective alternative for the treatment of RA.

## 1. Introduction

Autoimmune disorders are the consequence of the loss of ability of the immune system to differentiate between self- and non-self-antigens. Their incidence in the worldwide population is around 5% [1, 2]; these disorders are chronic and degenerative, being a major cause of disability resulting in an impact in the quality of life of the patients.

Dysregulation of several inflammatory pathways might be related to the pathogenesis of several autoimmune disorders, specifically immune-mediated inflammatory diseases (IMID). Although IMID occur in different organs or tissues, they seem to have in common those pathways where the tumor necrosis factor (TNF) is involved. TNF has been associated with rheumatoid arthritis (RA), psoriasis, psoriatic arthritis, and ankylosing spondylitis.

Nonresponding patients treated with nonsteroidal anti-inflammatory agents (NSAIDs), steroids, and common disease-modifying antirheumatic drugs (DMARDs) are prescribed with a newer class of DMARDs [3]. Recently, the development of novel DMARDs has been focused on specific TNF antagonists that block the interaction between TNF and its receptors. These biological agents include adalimumab, infliximab, certolizumab, golimumab, and etanercept, which were demonstrated to be more effective than traditional treatments in reducing the symptoms and preventing the progression of the disease [4].

Etanercept, in combination with methotrexate, has proved to be a successful treatment for RA [5]. Unlike monoclonal antibodies-TNF antagonists, etanercept is a recombinant dimeric fusion protein that contains two identical chains of the recombinant human TNF-receptor p75 monomer fused with a Fc domain of a human IgG1. This therapeutic protein was approved in 1998 by the Food and Drug Administration (FDA) as the first biologic response modifier (BRM) for the treatment of RA. It has also been prescribed for the treatment of other TNF-*α* mediated diseases [6]. The patent expiration date of the originator (in 2015 in Europe and 2028 in the US) has led to the development of etanercept's biosimilars in different countries. The advent of biosimilars will increase the health coverage, while improving the quality of life of patients that are unable to afford the cost of BMR therapies, especially in developing countries.

In order to assess the immunomodulatory activity comparability of biosimilar etanercept (Infinitam®) with respect to the reference product, we performed a study that included physicochemical and biological evaluations and a confirmatory pharmacodynamic clinical study in RA patients. All the studies presented herein were conducted in accordance with regulatory guidelines [7–9].

## 2. Materials and Methods

### 2.1. Materials

Biosimilar etanercept: Infinitam 25 mg vials were acquired from Probiomed S.A. de C.V., (Mexico, DF). Reference product: Enbrel® 25 mg vials were acquired from Amgen (Thousand Oaks, CA).

### 2.2. Physicochemical Properties

Identity was verified through tryptic peptide mappings analyzed by reverse phase ultra-performance-liquid-chromatography coupled to a tandem quadrupole/time-of-flight mass spectrometer (RP-UPLC-MS/MS). Three-dimensional structure was assessed by circular dichroism (CD) and fluorescence lifetime using the time correlated single photon counting technique (TCSPC). Heterogeneity was evaluated by intact mass by mass spectrometry (MS). Charge heterogeneity was assessed by capillary isoelectrofocusing (cIEF) of the whole molecule. Glycan microheterogeneity was studied using hydrophilic interaction ultra-performance-liquid-chromatography (HILI-UPLC). Sample treatment and analysis conditions were performed as previously described by Flores-Ortiz et al., 2014 [10] (MS, RP-UPLC-MS/MS, CD, and CEX-UPLC); Pérez Medina-Martínez et al., 2014 [11] (TCSPC); Espinosa-de la Garza et al. [12] (cIEF); and Miranda-Hernández et al., 2015 [13] (HILI-UPLC).

### 2.3. *In Vitro* Assay

Apoptosis inhibition assay was performed in U937 cells treated with TNF-*α* in the presence of different concentrations of etanercept. After 4 hours of treatment, Caspase 3/7-assay reagent was added and samples were incubated for 2–4 more hours. Luminescence emission was measured after 2–4 hours of incubation. The result is expressed as the ED_50_ value, calculated by four-parameter logistic curve fit using the Soft-MaxPro® software.

### 2.4. Clinical Study

A double-blinded, randomized, three-arm and prospective study was designed to evaluate the pharmacodynamic profile of etanercept. The three arms were combined, continuing the treatment with Infinitam after three cycles of treatment in order to evaluate interchangeability of Infinitam and the possible impact on the efficacy, as suggested by the Mexican health authorities.

The study protocol was approved by the Institutional Review Board/Independent Committee (IRB/IEC) of the participating research centers and by the Mexican health authorities. The study was conducted in accordance with the regulations and ethical principles stated on the Declaration of Helsinki, the principles of the International Conference on Harmonization (ICH), and the Guidelines for Good Clinical Practice (GCP). All patients signed an informed consent prior to the study; all procedures were explained in detail and all questions were resolved.

The aim of the study was to evaluate the biological effects of Infinitam compared to the reference product at 12 and 24 weeks of treatment with methotrexate therapy in patients with RA. Patients received either Infinitam or the reference product, according to their treatment group, at a dose of 25 mg twice a week by subcutaneous administration. 59 patients diagnosed with moderate to high degree RA were randomly assigned into three groups. Groups 1 and 3 were treated with Infinitam for 24 weeks. Conversely, group 2 was initially treated with the reference product for 12 weeks and then with Infinitam for the next 12 weeks. All patients received concomitant methotrexate. A three-month observational period was included after the completion of the treatments. Blood samples were collected from all patients for the determination of C-reactive protein (CRP), erythrocyte sedimentation rate (ESR), B-cell activating factor (BLyS), rheumatoid factor (RF), and TNF. Additionally Disease Activity Score (DAS28) was evaluated based on EULAR criteria with scores ranging from 0 to 9.4.

### 2.5. Statistical Analysis

Biological effect variables were compared with baseline values to those observed throughout the study in both treatment groups (group 2 versus group 1 + 3) to complete the 24 weeks of treatment. The statistical significance was *p* ≤ 0.05, two-tailed if appropriate unless otherwise noted. The analysis was performed with the SAS statistical package JMP®.

## 3. Results and Discussion

### 3.1. Physicochemical Analysis

**Figure 1:**
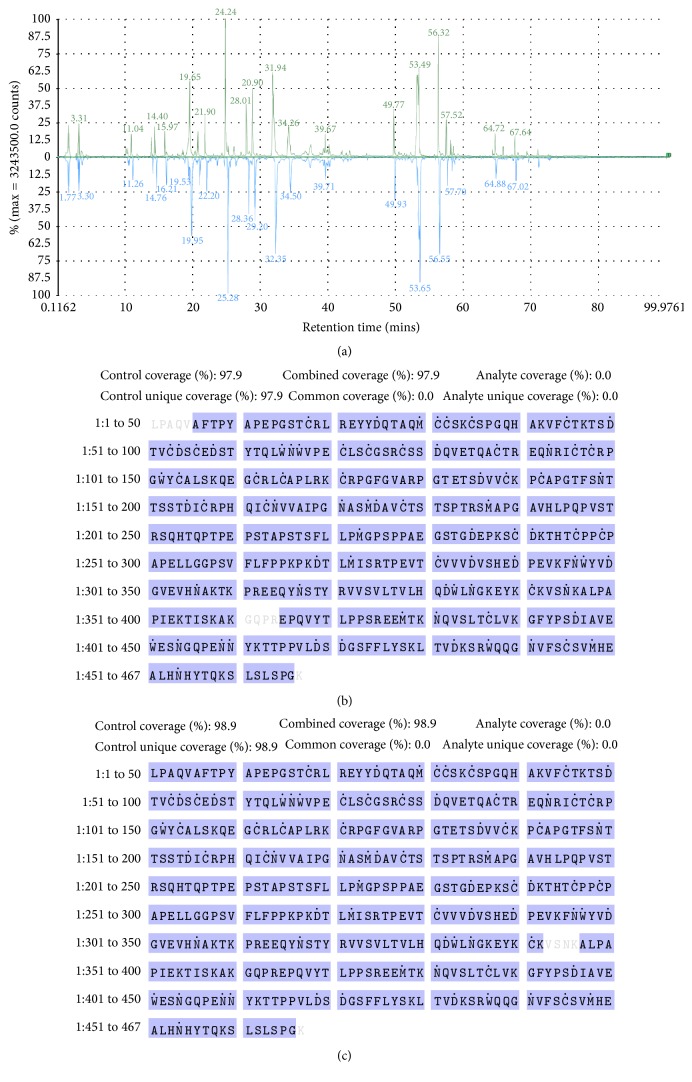
Confirmation of the primary structure by peptide mapping of (a) Infinitam (upper chromatogram in green) and the reference product (lower chromatogram in blue), and sequence coverages of (b) Infinitam and (c) the reference product with respect to the theoretical sequence of etanercept.

The first approach to ensure the adequate immunomodulatory response of biosimilar etanercept in the treatment of RA patients is to demonstrate the identity of the molecule. The protein's primary structure is essential to derive an appropriate higher-order structure, determined by amino acid side chains interactions influenced by the environment, allowing the exposure of the appropriate domains to recognize its target molecule: TNF alpha. Primary structure comparability was confirmed by the superimposition of peptide mapping chromatograms of Infinitam and the reference product (Figure 1(a)). The analysis was followed by the verification of the sequence coverage with respect to the theoretical sequence, being 98.3% and 97.0% for Infinitam and the reference product, respectively (Figures 1(b) and 1(c)). Overall, these results confirmed that both products contain etanercept as active pharmaceutical ingredient (API).

**Figure 2:**
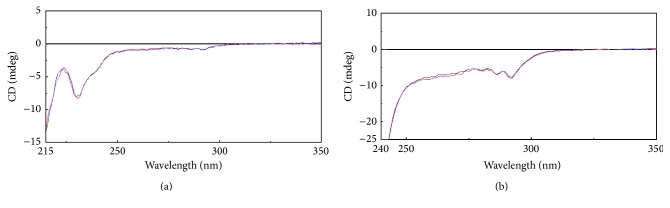
CD spectra of Infinitam (blue) and the reference product (red). (a) Far UV region and (b) near UV region.

Once the amino acid sequence of Infinitam was verified, spectroscopic techniques were used to compare the higher-order structure, as an indicator of an appropriate folding, between Infinitam and the reference product. For instance, CD analyses were performed to evaluate the secondary and tertiary structure. The obtained spectra in both far and near UV regions were superimposable (Figure 2) suggesting that both products possess comparable secondary and tertiary structures, respectively. On the other hand, the spatial disposition of the aromatic amino acids in etanercept, which is intrinsically correlated with its fluorescence lifetime (*τ*), was assessed by TCSPC [14–17]. The obtained results showed that the averaged *τ* of Infinitam was 1.56*E* − 09 ±0.02*E* − 10 s (*n* = 9, CI 95%), while the averaged *τ* for the reference product was 1.57*E* − 09 ±0.04*E* − 11 s (*n* = 9, CI 95%). Collectively CD and TCSPC analyses determined that the higher-order structure of Infinitam was comparable to the reference product. These results are supported by a previous report of the three-dimensional structure comparability between Infinitam and its reference product. Particularly, CD and TCSPC responses under native, denaturing, and denaturing-reduced conditions revealed similar structural features in both molecules, including their ordered and disordered regions that determine specific steric hindrances, as evidenced by the accessibility of free-thiols [11]. Accordingly, comparable target recognition is expected as long as charge heterogeneity ranges for both molecules overlap.

Furthermore, the evaluation of the biotherapeutic protein heterogeneity must be included to ensure that a biosimilar candidate possesses the same degree of heterogeneity with respect to the reference product. The inherent heterogeneity is the result of the protein's chemical and structural modifications that occur during its lifecycle, resulting in a group of closely related species (i.e., isoforms) that altogether constitute the protein's identity [18].

The heterogeneity in monoclonal antibodies has been widely characterized, studied, and correlated to the biological activity of the molecule [19–23]. Nonetheless, the heterogeneity of etanercept and its implications on the functionality of the molecule are still gaining knowledge.

**Figure 3:**
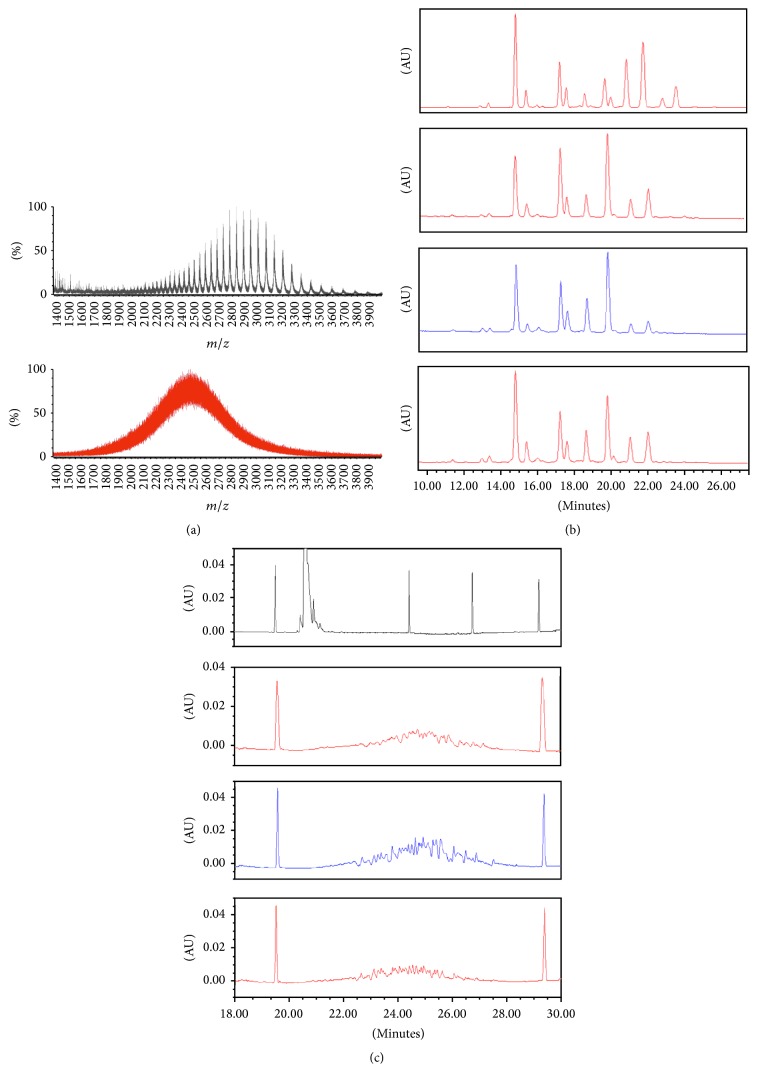
Heterogeneity analyses. (a) MS (*m*/*z*) analyses of etanercept (red) and rituximab (black), (b) glycan heterogeneity by HILI-UPLC of Infinitam (blue) and the reference product (red), and (c) charge heterogeneity by cIEF of Infinitam (blue), reference product (red), and rituximab (black).

Mass spectrometry analyses of intact molecule of etanercept and a monoclonal antibody evidenced a high degree of heterogeneity in the fusion protein in comparison to the monoclonal antibody (Figure 3(a)). As it can be observed, all the charge states of etanercept's isoforms cannot be resolved, developing a continuum profile. Thus, further analyses were performed in order to exhaustively characterize the heterogeneity of Infinitam in comparison to the reference product.

Charge heterogeneity of Infinitam and the reference product was evaluated through cIEF analysis (Figure 3(c)). The observed averaged pI values (weighted by isoform abundance) revealed a similar charge heterogeneity among products, being 5.50 ± 0.01 (*n* = 9, CI 95%) for Infinitam and 5.53 ± 0.32 (*n* = 9, CI 95%) for the reference product. Furthermore, pI isoforms ranged from 4.35 ± 0.07 to 6.57 ± 0.04 (*n* = 9, CI 95%) for Infinitam and 4.41 ± 0.20 to 6.68 ± 0.19 (*n* = 9, CI 95%) for the reference product, confirming similarity. It is worth to notice that typically pI ranges width is less than one pI unit for mAbs [13].

The glycan microheterogeneity of Infinitam and the reference product was also evaluated by HILI-UPLC since it is a relevant attribute on the immunomodulatory activity of biotherapeutic proteins. Chromatograms of different analyzed batches of the reference product showed several glycoforms with variable abundance (Figure 3(b)); Infinitam glycoforms lied within this wide heterogeneity, revealing comparability. It its worth to mention that for monoclonal antibodies, it has been reported that specific glycan isoforms could affect the affinity to the receptors involved in their effector functions and stability due to charge and steric hindrances [20, 22–25]. However, for fusion proteins, the impact on the global charge, stability, and steric hindrances of specific glycan isoforms need to be assessed, either experimentally or theoretically [26], considering that, for etanercept, those glycans do not lie within the recognition sites of its target molecule. In this sense, the demonstration of a similar biological activity supports that the observed heterogeneity in Infinitam constitutes a basis for a biosimilar as efficient as the reference product.

### 3.2. Biological Characterization

The assessment of the biological activity, after the physicochemical characterization, confirms that the analyzed product has the same identity, higher-order structure, and posttranslational modifications as the reference product. The potency assay evaluated the ability of etanercept to prevent the interaction of TNF with cellular TNFR and can be used as a first indicator of its pharmacological activity.

**Figure 4:**
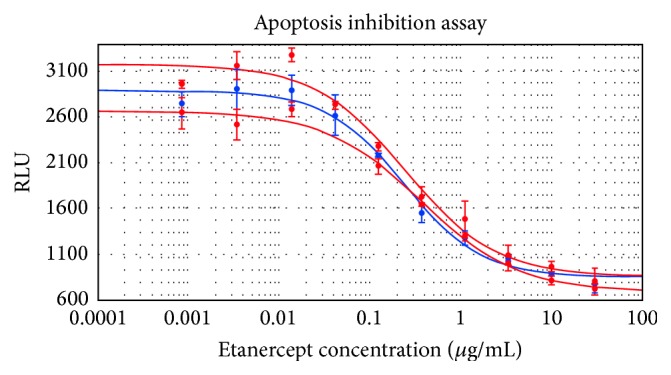
Dose-response curves of Infinitam (blue) and the reference product (red).

The results confirmed a comparable neutralization of TNF*α* activity between Infinitam and the reference product (Figure 4), thus reducing the uncertainty of presenting different pharmacodynamics profiles. In this regard, the relative content of all etanercept isoforms (i.e., acidic, basic, and glycan isoforms) seems to be determinant for a comparable clinical profile. However, further studies are required to understand the direct correlation between specific physicochemical properties (charge, glycosylation) and their impact on the pharmacological behavior of etanercept. Accordingly, a clinical assessment was performed to show a comparable modulation of the inflammatory response with Infinitam and the reference product.

### 3.3. Clinical Assessment

**Figure 5:**
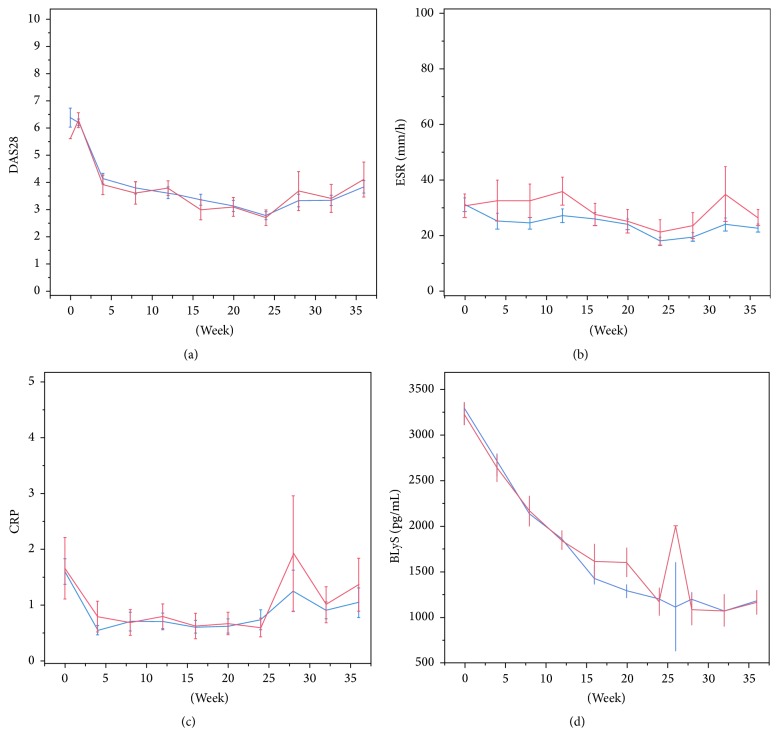
Pharmacodynamic parameters of groups treated with Infinitam (blue) and the reference product (red). (a) DAS28 per visit, (b) ESR per visit, (c) CRP per visit, and (d) BLyS per visit.

The comparison of the biological effect of methotrexate-associated Infinitam and reference product in patients with RA was evaluated based on the seric levels of the BLyS protein, ESR, CRP, TNF, and RF. Additionally, efficacy was assessed by the response of the DAS28 using EULAR criteria [27] at 12 and 24 weeks of treatment. DAS28 consist in the number of painful and swollen joints, ESR, and overall disease activity. The results showed that 70% of the patients had a reduction of at least 1.2 points and reached a DAS28 score lower than 3.2, representing a moderate response on disease activity (Figure 5(a)).

ESR is an indicator of inflammation whose value is increased because of various factors, including RA. This indicator is useful to assess the treatment response based on EULAR criteria. A reduction was observed in all patients in ESR levels in comparison with their ESR basal values (Figure 5(b)). The results showed a similar behavior between groups at 12 and 24 weeks (*p* = 0.116 and *p* = 0.389, resp.).

CRP is also an indicator of inflammation. There are few factors that modify production levels of CRP besides liver failure. Overall, there was a reduction in CRP levels, maintained at weeks 12 and 24, when compared to basal levels. In spite of the values dispersion, a trend towards improvement on acute inflammation of the affected joints was observed till the end of the study. Finally, increasing CRP levels in all treatment groups during the observational period should be a consequence of etanercept depletion (Figure 5(c)).

BLyS is an important protein for regulatory functions in survival, maturation, and differentiation of B cells. It has been reported that after initiating therapy with anti-TNF drugs, patients with RA whose plasma levels of BLyS protein are reduced have a better prognosis than those patients whose BLyS levels are not reduced. The reduction of BLyS levels in both groups was statistically significant and similar in both groups of treatment (*p* = 0.946 and *p* = 0.865, weeks 12 and 24) (Figure 5(d)).

Serum levels of TNF showed an upward trend, since etanercept prevents the association to the TNF-R and its further internalization and degradation, thereby increasing the circulating levels of this cytokine. Accordingly, no statistically significant differences between treatment groups at weeks 12 and 24 were observed (*p* = 0.178 and *p* = 0.178, resp.).

Finally, as a diagnostic measure, levels of RF were part of the initial evaluation of patients with RA included in the protocol. Although there is no consensus of the correlation levels with the disease status, it is well known that a modification of RF could be used as a biomarker of treatment response.

The observed results can be explained by the inhibition effect on TNF on both Infinitam and the reference product. The clinical response was rapidly achieved within the first four weeks of treatment. The behavior of the two products containing etanercept was similar. The tendency of clinical response can be considered satisfactory according to data published by other authors [28, 29].

## 4. Conclusions

The physicochemical and biological characterization studies revealed no differences in the identity and higher-order structure between Infinitam and the reference product. Regarding etanercept's heterogeneity a major diversity of charge and glycan isoforms was observed, even among batches of the reference product. For this reason, the establishment of acceptable ranges for these isoforms content is still unclear, since no significant effect was observable in the immunomodulatory activity of etanercept during biological assays. Therefore, a narrowed clinical study capable of demonstrating and confirming that both products have similar immunomodulatory response becomes critical. Altogether, the physicochemical, biological, and clinical comparability studies resulted in a similar immunomodulatory activity between the evaluated products.
